# Supplementary figures and images for: Development of an in vivo syngeneic mouse transplant model of invasive intestinal adenocarcinoma driven by endogenous expression of Pik3caH1047R and Apc loss
Source: PLoS One. 2024 Aug 2;19(8):e0308051. doi: 10.1371/journal.pone.0308051 (PMC11296624; doi:10.1371/journal.pone.0308051)

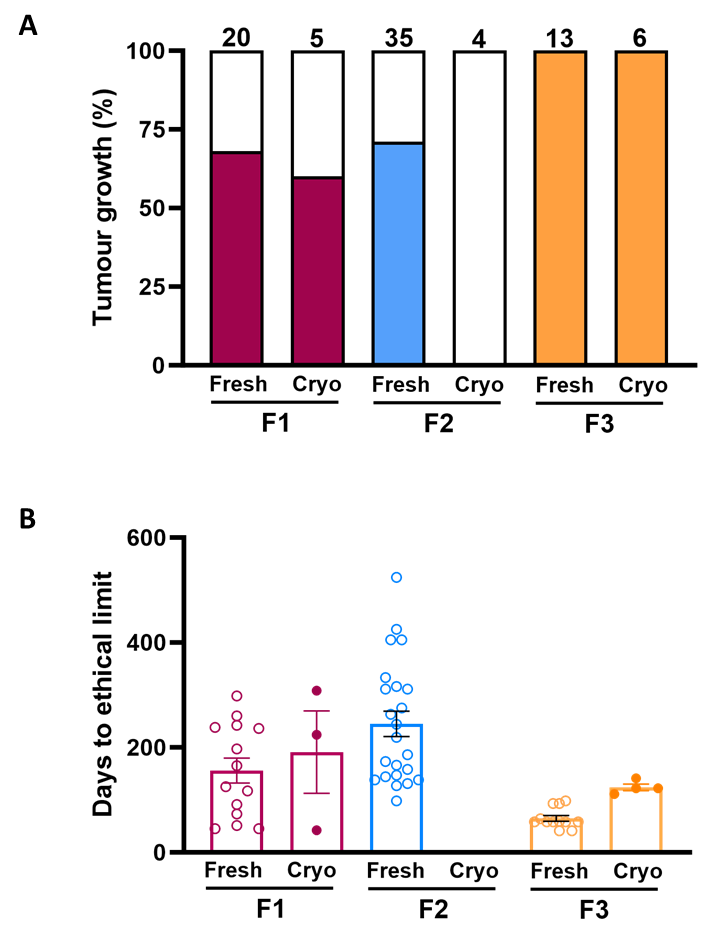

Supplement: S1 Fig — A. Stacked bar graphs display percentage of transplanted fresh and cryopreserved (Cryo) tumour tissues that generated new tumour growth. Magenta, blue, and orange shading represents the proportion of F1, F2 and F3 tumours, respectively, that grew upon transplantation. White represents the proportion of tumours that did not grow. Total numbers transplanted (n) are shown at the top of each bar. F1 and F2 tumours were transplanted intramuscularly whereas F3 tumours were transplanted subcutaneously. B. The number of days taken for tumours to reach ethical limit across each generation of transplantations, comparing fresh and cryopreserved tumour tissue. Magenta = F1, Blue = F2, Orange = F3. Open circles represent fresh tissue, filled circles represent cryopreserved tissue. Boxes represent the mean. Error bars represent SEM. (TIF) [file pone.0308051.s001.tif]
