# Supplementary material for: Development of an in vivo syngeneic mouse transplant model of invasive intestinal adenocarcinoma driven by endogenous expression of Pik3caH1047R and Apc loss
Source: PLoS One. 2024 Aug 2;19(8):e0308051. doi: 10.1371/journal.pone.0308051 (PMC11296624; doi:10.1371/journal.pone.0308051)
Supplement: S1 Raw images — (PDF) [file pone.0308051.s002.pdf]

Original image of PCR blot shown in Figure 2B

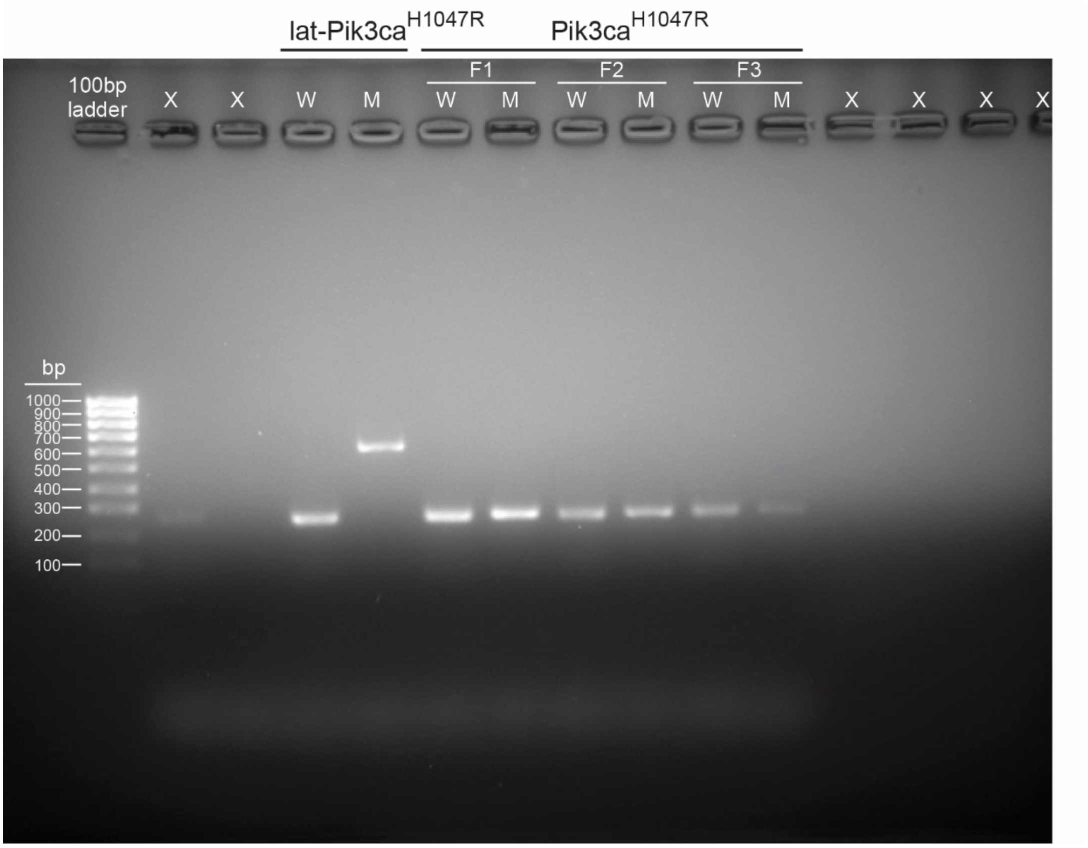

X = empty or non-relevant lane cropped out for clarity.

W = Wildtype

M = Mutant
