# Supplementary material for: Development of an in vivo syngeneic mouse transplant model of invasive intestinal adenocarcinoma driven by endogenous expression of Pik3caH1047R and Apc loss
Source: PLoS One. 2024 Aug 2;19(8):e0308051. doi: 10.1371/journal.pone.0308051 (PMC11296624; doi:10.1371/journal.pone.0308051)
Supplement: Attachment — Submitted filename: Response to reviewers.docx [file pone.0308051.s003.docx]

Gianpaolo Papaccio, M.D., Ph.D.

Academic Editor

PLOS ONE

Dear Dr. Papaccio,

Thank you for your consideration of our manuscript “Development of an in vivo syngeneic mouse transplant model of invasive intestinal adenocarcinoma driven by endogenous expression of *Pik3ca*^H1047R^ and *Apc* loss” by de la Heras *et al*. which we submitted for publication in PLOS ONE as a research article.

We are pleased that you found our manuscript interesting and well performed and are grateful for the opportunity to address the reviewer’s, and your, comments.

Please find our response below.

**Response to reviewer and editor comments**

We thank the reviewer and editor for their positive comments and suggestions.

As suggested by the reviewer, we have added some comments to the discussion to better highlight the relevance to human cancer and how we feel our model could be exploited in future studies (lines 315-332 in tracked version).

Regarding evaluating the relationships with TME, we agree that this is a particularly interesting question which our model will be very useful for addressing, and we have now added some comments to this effect in the discussion (lines 326-332 in tracked version). However, we do feel that such studies are beyond the scope of our current manuscript which simply aims to report our model.

Indeed, we would like to stress this manuscript is intended to simply report the development of a novel syngeneic mouse model of a *PIK3CA*-driven colorectal cancer. We have not sought to address any specific biological or clinical question in this manuscript. Nevertheless, we believe this model will be a valuable adjunct to current models and we feel it important to report this model to the wider research community in a timely fashion.

We note that the reviewer and editor request that we cite several specific papers. We have read these articles with interest.

**Papaccio et al. (2023) J Exp Clin Cancer Res. 42(1):8**. This paper describes the generation of a large number of patient-derived organoid models that they use for a proteotranscriptomic study to explore the mechanisms underlying drug response. Although they use human organoids, and an *in vitro* cell culture system, we agree that it is an excellent example of how our model could be exploited and have now included reference to this paper in our discussion (reference 22, line 326 of tracked change version).

**Papaccio et al. (2020) Cancers. 12: 3611**. This is a review article that discusses two potentially practice-changing clinical trials and how they may affect our understanding of treating locally advanced rectal cancers. An interesting and informative article but unclear how it specifically relates to our current manuscript.

**Tarazona et al. (2020) ESMO Open. 5: e000847**. A clinical study of ctDNA and CDX2 expression as markers of recurrence in patients with localised colon cancer but of unclear relevance to our manuscript.

**Papavassiliou et al. (2023) Int J Mol Sci. 24; 17101**. An editorial article discussing the targeting the TGF-β signalling axis in metastatic colorectal cancer. Again, the specific relevance to our current manuscript is unclear to us.

While we agree that these are important and noteworthy articles, and may arguably be tangentially related to our manuscript, given our model is of colorectal cancer, we would contend that they address topics that are largely outside the scope of our current manuscript. If citing these articles is essential for acceptance, we would respectfully request some further guidance as to what is specifically required and clarification as to why these particular articles should be cited.

**Tarazona et al. (2020) ESMO Open. 5: e000847**. A clinical study of ctDNA and CDX2 expression as markers of recurrence in patients with localised colon cancer but of unclear relevance to our manuscript.

**Papavassiliou et al. (2023) Int J Mol Sci. 24; 17101**. An editorial article discussing the targeting the TGF-β signalling axis in metastatic colorectal cancer. Again, the specific relevance to our current manuscript is unclear to us.

While we agree that these are important and noteworthy articles, and may arguably be tangentially related to our manuscript, given our model is of colorectal cancer, we would contend that they address topics that are largely outside the scope of our current manuscript. If citing these articles is essential for acceptance, we would respectfully request some further guidance as to what is specifically required and clarification as to why these particular articles should be cited.

We thank you for considering this revised manuscript and trust you will now find it acceptable for publication in PLOS ONE.

We look forward to your response.

Yours sincerely,

**Prof. Wayne A. Phillips PhD** (for, and on behalf of, all the authors)

Department of Cancer Research

Email: [wayne.phillips@petermac.org](mailto:wayne.phillips@petermac.org)
